# Sub-Nyquist SAR Imaging and Error Correction Via an Optimization-Based Algorithm

**DOI:** 10.3390/s24092840

**Published:** 2024-04-29

**Authors:** Wenjiao Chen, Li Zhang, Xiaocen Xing, Xin Wen, Qiuxuan Zhang

**Affiliations:** 1 The Department of Space Control and Communications, Space Engineering University, Beijing 102249, China; chenwenjiao008@163.com (W.C.); xxc012345@163.com (X.X.); wx19951017@126.com (X.W.); zhangqiuxuan@yeah.net (Q.Z.); 2 The 15th Research Institute of China Electronics Technology Corporation, Beijing 100083, China

**Keywords:** sub-Nyquist synthetic aperture radar (SAR) imaging, error correction, optimization-based algorithm, Bayesian estimation

## Abstract

Sub-Nyquist synthetic aperture radar (SAR) based on pseudo-random time–space modulation has been proposed to increase the swath width while preserving the azimuthal resolution. Due to the sub-Nyquist sampling, the scene can be recovered by an optimization-based algorithm. However, these methods suffer from some issues, e.g., manually tuning difficulty and the pre-definition of optimization parameters, and a low signal–noise ratio (SNR) resistance. To address these issues, a reweighted optimization algorithm, named pseudo-ℒ_0_-norm optimization algorithm, is proposed for the sub-Nyquist SAR system in this paper. A modified regularization model is first built by applying the scene prior information to nearly acquire the number of nonzero elements based on Bayesian estimation, and then this model is solved by the Cauchy–Newton method. Additionally, an error correction method combined with our proposed pseudo-ℒ_0_-norm optimization algorithm is also present to eliminate defocusing in the motion-induced model. Finally, experiments with simulated signals and strip-map TerraSAR-X images are carried out to demonstrate the effectiveness and superiority of our proposed algorithm.

## 1. Introduction

High-resolution wide-swath (HRWS) synthetic aperture radar (SAR) provides a short repeat cycle so that it has a high-efficiency acquisition capability [1,2]. Although the azimuthal multi-channel SAR [3] and multi-input multi-output (MIMO) SAR [4] can achieve a high azimuthal resolution and wide-range swath, it has a large amount of data and a large antenna. As the compressive sensing (CS) theorem posits [5,6,7], an innovative system concept called sub-Nyquist SAR based on pseudo-random time–space modulation breaks the limitation of the Nyquist sampling theorem with a single channel [8]. Simultaneously, the spatial and temporal phase modulation based on the optimization-based algorithm guarantees azimuthal resolution and wide-swath coverage mosaicked by several range sub-swaths. It adopts sub-Nyquist sampling along the azimuthal dimension, and the observed scene is recovered by sub-Nyquist SAR imaging [8].

In spite of the sub-Nyquist SAR achieving the above merits, sub-Nyquist SAR imaging still has two limitations, as follows:

- Under the assumption of satisfying restricted isometry property (RIP) [9], CS algorithms are vital to sub-Nyquist SAR imaging and include greedy algorithms [10,11], the ℒ_1_-norm optimization algorithms [12,13], and Bayesian-based methods [14,15,16], where the ℒ_1_-norm optimization algorithm has a better performance in terms of the recovered error evaluated by the mean square error (*MSE*) [17,18,19]. Although the ℒ_1_-norm optimization algorithm has achieved a better-recovered performance, these methods still suffer from some problems, i.e., manually tuning difficulty and the pre-definition of optimization parameters (e.g., regularization parameter and thresholding parameter), and a low signal–noise ratio (SNR) resistance. However, these CS methods do not take full advantage of the scene prior information that we may hold, and sparse property is imposed uniformly and independently on each variable. Some low signal–noise ratio (SNR) targets in the sparse scene cannot be accurately recovered and it often yields false targets by the ℒ_1_-based method. Although some reweighted optimization-based algorithm has already been proposed [20,21], there is still no knowledge of how and why to select an approximately fair rule in sub-Nyquist SAR imaging to further mitigate the impact of empirical parameter setting on reconstructed performance.
- In addition, the imaging process requires the knowledge of motion parameters, e.g., radar position and radar equivalent velocity [5]. However, the radar platform may deviate from the pre-defined track, and the equivalent velocity is estimated by the curve-fitting method or approximate expression in a practical application [22]; uncertainties and errors may be introduced into the motion-induced model so that the recovered scene may defocus to decrease the image quality [23,24,25,26]. A technology called auto-focusing removes these phase errors [27]. In recent years, many sparsity-driven algorithms [28,29,30,31,32,33,34] have been proposed to solve the defocusing problem and achieve an effective performance. However, the references [28,29,30,31,32,33] do not fully formulate the motion error and adopt an approximate expression so that the error is not removed. The reference [34] integrated the deep SAR imaging algorithm to remove the motion error.

In this paper, we propose a pseudo-ℒ_0_-norm optimization algorithm based on Bayesian estimation to further improve the sub-Nyquist SAR imaging performance. The proposed algorithm penalizes the regularization item with the scene prior information, i.e., the reciprocal of its previous solution, to nearly acquire the number of nonzero values. Since this method approximates a ℒ_0_-based algorithm, which needs to be minimized in the sparse recovery, we named it a pseudo-ℒ_0_-norm optimization algorithm. Sub-Nyquist SAR imaging includes three steps: range compression, range cell migration correction (RCMC), and azimuth compression [5]. The traditional matched filtering (MF) method is adopted for range compression. After RCMC, a pseudo-ℒ_0_-norm optimization algorithm is adapted to achieve azimuth compression. This method not only takes advantage of the scene prior information but also establishes an approximately fair penalized rule so that it can recover low SNR targets and remove false targets compared to the ℒ_1_-norm optimization algorithm. In addition, an error correction method integrated with a pseudo-ℒ_0_-norm optimization algorithm eliminates the influence of phase error and improves the image quality. This method includes two steps: scene recovery based on a pseudo-ℒ_0_-norm optimization algorithm and error estimation by minimizing the least-squares target function. The two steps are successively iterative and the recovered matrix is updated according to the estimated error. For the exact expression of error is not fully formulated, we use the random phase error regardless of the error expression. The numerical simulation results are detailed in the following sections to make an evident advantage of the proposed algorithm.

The rest of this paper is organized as follows. In Section 2, the observation model for the sub-Nyquist SAR based on the pseudo-random time–space modulation is first described. Then, we build a pseudo-ℒ_0_-norm regularization model based on Bayesian estimation, and this regularization model can be solved by the Cauchy–Newton method. Additionally, an error correction method integrated with a pseudo-ℒ_0_-norm optimization algorithm is proposed to remove errors and eliminate defocusing. Simulation experiments and data experiments with real TerraSAR-X images confirm the effectiveness and superiority of our proposed method, presented in Section 3. In Section 4, the discussion shows the performance and advantages of our proposed algorithm. Section 5 concludes this paper.

## 2. Materials and Methods

In this section, the observation model of the sub-Nyquist SAR based on the pseudo-random time–space modulation is described. To further improve the sub-Nyquist SAR imaging performance, a pseudo-ℒ_0_-norm regularization model is built and solved by the Cauchy–Newton method to obtain a pseudo-ℒ_0_-norm optimization algorithm. In addition, an error correction method integrated with our proposed pseudo-ℒ_0_-norm optimization algorithm is proposed to eliminate the effect of the phase error and achieve autofocusing.

### 2.1. Sub-Nyquist SAR Imaging and Error Correction Signal Models Based on Pseudo-Random Time–Space Modulation

#### 2.1.1. Sub-Nyquist SAR Imaging Model

**Figure 1:**
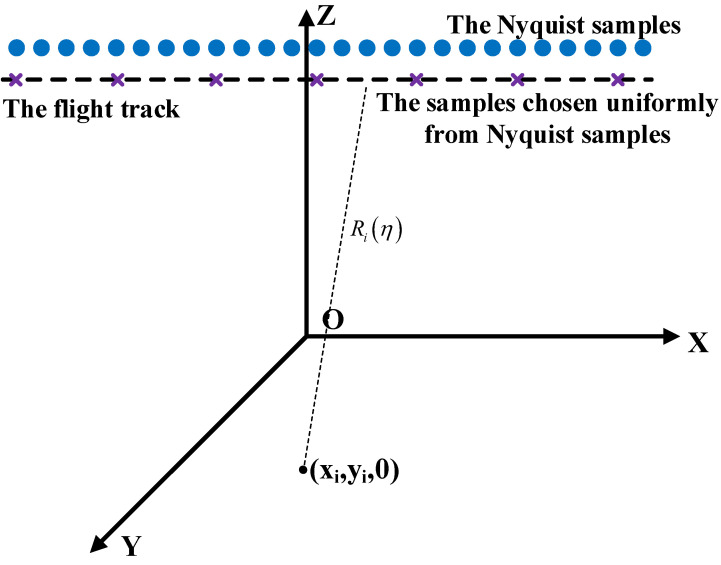
The imaging geometry of the sub-Nyquist SAR. 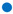 denotes the Nyquist samples. 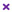 demonstrates the real azimuthal samples chosen uniformly from Nyquist samples in the sub-Nyquist SAR system based on the pseudo-random time–space modulation.

For traditional HRWS systems, e.g., the azimuthal multi-channel SAR [3] and MIMO SAR [4], the equivalent sampling still satisfies the Nyquist theorem. To lower the amount of data and break the conflict between high resolution and wide swath, the sub-Nyquist SAR based on pseudo-random time–space modulation has been proposed [8]. The imaging geometry of the sub-Nyquist SAR is shown in Figure 1, where η is slow time along the azimuth and $R_{i}\left(\eta \right)$ represents the range between the radar and the point target located at the coordinate $\left(x_{i},y_{i},0\right)$ at the azimuth time η. $x_{i}$ and $y_{i}$ denote the azimuth and range coordinates, respectively. Because a two-dimensional image, i.e., azimuth and range, is considered, the coordinate $\left(x_{i},y_{i},0\right)$ is simplified as $\left(x_{i},y_{i}\right)$. 

Similarly to the traditional SAR system, the raw data in the sub-Nyquist SAR for many point targets can be written as:

(1) $$s_{c}\left(\tau ,\eta \right)=\sum_{x_{i},y_{i}}\sigma_{i}W_{i}\left(\tau ,\eta \right)\exp\left\{j\pi K_{r}{\left[\tau -2R_{i}\left(\eta \right)/c\right]}^{2}\right\}\cdot \exp\left\{-j4\pi R_{i}\left(\eta \right)/\lambda \right\}\cdot \exp\left\{j\varphi_{i}\left(\eta \right)\right\}+n\left(\tau ,\eta \right)$$

where τ is the fast time along the range dimension, i.e., the sampling moment during one pulse width. η is the slow time along the azimuth dimension, i.e., the moment of the transmitting pulse. $\sigma_{i}$ and $W_{i}\left(\tau ,\eta \right)$ are the backscattering coefficient and the weighting pattern corresponding to the *i*-th target at $\left(x_{i},y_{i}\right)$, respectively. $\varphi_{i}\left(\eta \right)$ is the random phase based on the pseudo-random time–space modulation corresponding to the target $\left(x_{i},y_{i}\right)$. $K_{r}$ denotes the chirp rate of the linear frequency-modulated (LFM) signal, c is the light speed, λ is the wavelength, and $n\left(\tau ,\eta \right)$ is the system noise.

To relieve the inherent contradiction in the HRWS system, sub-Nyquist sampling in the sub-Nyquist SAR is implied along the azimuthal dimension, and the sampling method is as shown in Figure 1. The sub-Nyquist SAR system only adopts the traditional MF method to not recover the scene exactly [5]. Accordingly, sub-Nyquist SAR imaging includes three steps: (1) range compression based on MF; (2) range cell migration correction (RCMC): interpolate by zero padding every azimuthal signal after range compression and then sum up all the range-compressed signal of grids on the same range cell to the pre-defined grid; and (3) azimuth reconstruction with sub-Nyquist samples based on the CS algorithm. After implementing range compression and RCMC to Equation (1), the signal at a certain range cell is represented by:

(2) $$\begin{array}{l} s_{c}\left(\tau_{0},\eta \right)\\ =\sum_{x_{i},y_{i}}\sigma_{i}W_{i}\left(\tau_{0},\eta \right)T_{r}\sin c\left\{K_{r}T_{r}\left(\tau_{0}-2\frac{R_{i}\left(\eta -\eta_{ci}\right)}{c}\right)\right\}\cdot \exp\left\{-j4\pi \frac{R_{i}\left(\eta -\eta_{ci}\right)}{\lambda }\right\}\cdot \exp\left\{j\varphi_{i}\left(\eta \right)\right\}+n\left(\tau_{0},\eta \right) \end{array}$$

where $\eta_{ci}$ is the beam center crossing time for the target $\left(x_{i},y_{i}\right)$. $T_{r}$ is the pulse width and $\sin c\left(\cdot \right)$ denotes the *sinc* function.

Let $\sigma ={\left[\sigma_{1},\sigma_{2},\cdots ,\sigma_{M}\right]}^{T}$ be the vectorized backscattering cross-sections of targets on the same range cell, and $s_{N\times 1}=[s_{c}\left(\tau_{0},\eta_{1}\right),s_{c}\left(\tau_{0},\eta_{2}\right),\cdots ,s_{c}\left(\tau_{0},\eta_{N}\right){]}^{T}$ be the vectored signal after range compression and RCMC; then,

(3) $$s_{N\times 1}=D_{N\times M}\sigma_{M\times 1}+n_{N\times 1}$$

where N is the sampling number on the azimuthal dimension and M is the number of resolution cells at the certain range cell in the observed scene. $D_{N\times M}={\left\{D_{i}\left(\tau_{0},\eta_{n}\right)\right\}}_{n=1,i=1}^{N,M}$ denotes the mapping relation between the received signal and the scene, and $D_{i}\left(\tau_{0},\eta_{n}\right)=W_{i}\left(\tau_{0},\eta_{n}\right)\cdot T_{r}\cdot \exp\left\{-j4\pi R_{i}\left(\eta_{n}-\eta_{ci}\right)/\lambda \right\}\cdot \exp\left\{j\varphi_{i}\left(\eta \right)\right\}$ $n_{N\times 1}={\left[n\left(\tau_{0},\eta_{1}\right),n\left(\tau_{0},\eta_{2}\right),\cdots ,n\left(\tau_{0},\eta_{N}\right)\right]}^{T}$ is the noise.

#### 2.1.2. Motion Error Model

The recovered scene $\sigma_{M\times 1}$ can be estimated by Equation (3), while the recovered matrix $D_{N\times M}$ can exactly reflect the relation between the raw data and the recovered scene. However, uncertainties and errors exist in the matrix $D_{N\times M}$, and the inaccuracy of the motion-induced model leads to the phase error [22]. If the preset matrix $D_{N\times M}$ in Equation (3) is still used to recover the scene $\sigma_{M\times 1}$ without extra processing steps, it may cause the defocusing of the reconstructed scene. A technology called autofocusing removes these phase errors.

**Figure 2:**
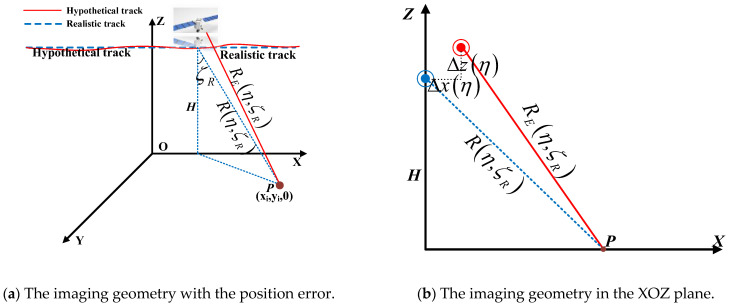
The imaging geometry with the position error. The solid line denotes the realistic track, and the dashed line denotes the hypothetical track. $R\left(\eta ,\zeta_{R}\right)$ and $R_{E}\left(\eta ,\zeta_{R}\right)$ are the hypothetical slant range and real slant range with the position error, respectively. $\zeta_{R}$ is the pitch angle. ***P*** is the target point. H is the orbital height. Figure (**b**) is the projection of target point ***P*** on the XOZ plane. $\Delta x\left(\eta \right)$ and $\Delta z\left(\eta \right)$ are the range between the realistic position and the hypothetical position on the x-axis and z-axis, respectively.

To solve the problem of error correction, it is important to establish the exact mapping model so that we need to discern the uncertain factors. We mainly considered the position error and velocity error leading to the phase error in this paper. The SAR satellite is affected by different perturbations during on-orbit flying; so, it may cause the position error, as demonstrated in the following Figure 2, and the velocity error of the satellite. Firstly, we analyzed the position errors. The position error is mainly the deviation between the realistic position and the hypothetical position on the x-axis and z-axis. In Figure 2a, the solid line denotes the realistic track, and the dashed line denotes the hypothetical track. The hypothetical slant range between the radar and target without position error is:

(4) $$R\left(\eta ,\zeta_{R}\right)=\sqrt{{\left(\frac{H}{\cos\zeta_{R}}\right)}^{2}+{\left(V_{e}\eta -V_{e}\eta_{0}\right)}^{2}}\approx \frac{H}{\cos\zeta_{R}}+\frac{{V_{e}}^{2}\cos\zeta_{R}}{2H}{\left(\eta -\eta_{0}\right)}^{2}$$

where $V_{e}$ is the radar velocity. $\eta_{0}$, the Doppler center moment, is the azimuthal moment when the radar is the nearest to the target.

When a position error exists, the real slant range $R_{\xi }\left(\eta ,\zeta_{R}\right)$ between the radar and target is:

(5) $$\begin{array}{ll} R_{\xi }\left(\eta ,\zeta_{R}\right) & =\sqrt{{\left(H\tan\zeta_{R}-\Delta x\left(\eta \right)\right)}^{2}+{\left(V_{e}\eta -V_{e}\eta_{0}\right)}^{2}+{\left(H+\Delta z\left(\eta \right)\right)}^{2}}\\ & \approx \frac{H}{\cos\zeta_{R}}+\frac{{V_{e}}^{2}\cos\zeta_{R}}{2H}{\left(\eta -\eta_{0}\right)}^{2}+\Delta z\left(\eta \right)\cdot \cos\zeta_{R}-\Delta x\left(\eta \right)\cdot \sin\zeta_{R}\\ & \approx R\left(\eta ,\zeta_{R}\right)+\Delta R_{\xi }\left(\eta ,\zeta_{R}\right) \end{array}$$

$\Delta R_{\xi }\left(\eta ,\zeta_{R}\right)=\Delta z\left(\eta \right)\cdot \cos\zeta_{R}-\Delta x\left(\eta \right)\cdot \sin\zeta_{R}$ is the position error. Regardless of the weight $W_{i}\left(\tau_{0},\eta_{n}\right)\cdot T_{r}$ in Equation (2), the realistic echo signal $s_{error}\left(\eta ,\zeta_{R}\right)$ of target point ***P*** at a certain range cell after range compression and RCMC is denoted as

(6) $$\begin{array}{ll} s_{error}\left(\eta ,\zeta_{R}\right) & =\sigma_{P}\cdot \exp\left\{-j\frac{4\pi }{\lambda }R_{\xi }\left(\eta ,\zeta_{R}\right)\right\}\\ & =\sigma_{P}\cdot \exp\left\{-j\frac{4\pi }{\lambda }\left(R\left(\eta ,\zeta_{R}\right)+\Delta R_{\xi }\left(\eta ,\zeta_{R}\right)\right)\right\}\\ & =s_{0}\left(\eta ,\zeta_{R}\right)\cdot H_{\Delta R}\left(\eta ,\zeta_{R}\right) \end{array}$$

where $s_{0}\left(\eta ,\zeta_{R}\right)=\sigma_{P}\cdot \exp\left\{-j4\pi R\left(\eta ,\zeta_{R}\right)/\lambda \right\}$ is the raw signal without the position error. $H_{\Delta R}\left(\eta ,\zeta_{R}\right)$ is the position error signal.

Secondly, we analyzed the mathematical model for the velocity error. Regardless of the weight $W_{i}\left(\tau_{0},\eta_{n}\right)\cdot T_{r}$ in Equation (2), the realistic echo signal $s_{error}\left(\eta ,V_{Ee}\right)$ of target point ***P*** at a certain range cell after range compression and RCMC is:

(7) $$s_{error}\left(\eta ,V_{Ee}\right)=\sigma_{P}\cdot \exp\left\{-j\frac{4\pi R_{\xi }\left(\eta ,V_{Ee}\right)}{\lambda }\right\}$$

where $V_{Ee}$ is the equivalent velocity. The raw signal is approximate to the linear frequency modulation signal and is written as:

(8) $$s_{error}\left(\eta ,V_{Ee}\right)=\exp\left\{f_{d}\eta +f_{r}\left(V_{Ee}\right)\eta^{2}\right\}$$

where $f_{d}$ is the Doppler center frequency and the Doppler modulated rate is $f_{r}\left(V_{Ee}\right)=2{V_{Ee}}^{2}\cos^{3}\zeta_{A}/\lambda R$, and $\zeta_{A}$ is the squint angle on the azimuthal dimension. When the hypothetical equivalent velocity $V_{E}$ exists, $f_{r}\left(V_{Ee}\right)$ is unfolded at $V_{Ee}=V_{E}$:

(9) $$f_{r}\left(V_{Ee}\right)=f_{r}\left(V_{E}\right)+{f_{r}}^{\prime }\left(V_{E}\right)\left(V_{Ee}-V_{E}\right)+\frac{1}{2!}{f_{r}}^{\prime \prime }\left(V_{E}\right){\left(V_{Ee}-V_{E}\right)}^{2}+\cdots +\frac{1}{n!}{f_{r}}^{\left(n\right)}\left(V_{E}\right){\left(V_{Ee}-V_{E}\right)}^{n}+\cdots$$

If the error caused by the equivalent velocity is small, the above equation besides the first item $f_{r}\left(V_{E}\right)$ is also small and is denoted as $o_{r}\left(V_{Ee}\right)$:

(10) $$o_{r}\left(V_{Ee}\right)={f_{r}}^{\prime }\left(V_{E}\right)\left(V_{Ee}-V_{E}\right)+\frac{1}{2!}{f_{r}}^{\prime \prime }\left(V_{E}\right){\left(V_{Ee}-V_{E}\right)}^{2}+\cdots +\frac{1}{n!}{f_{r}}^{\left(n\right)}\left(V_{E}\right){\left(V_{Ee}-V_{E}\right)}^{n}+\cdots$$

Substituting (24) and (25) into (23),

(11) $$\begin{array}{ll} s_{error}\left(\eta ,V_{Ee}\right) & =\exp\left\{f_{d}\eta +f_{r}\left(V_{Ee}\right)\eta^{2}\right\}=\exp\left\{f_{d}\eta +f_{r}\left(V_{E}\right)\eta^{2}+o_{r}\left(V_{Ee}\right)\eta^{2}\right\}\\ & =\exp\left\{f_{d}\eta +f_{r}\left(V_{E}\right)\eta^{2}\right\}\cdot \exp\left\{o_{r}\left(V_{Ee}\right)\eta^{2}\right\}\\ & =s_{0}\left(\eta ,V_{E}\right)\cdot \exp\left\{o_{r}\left(V_{Ee}\right)\eta^{2}\right\}=s_{0}\left(\eta ,V_{E}\right)\cdot H_{V_{Ee}}(\eta ) \end{array}$$

where $s_{0}\left(\eta ,V_{E}\right)=\exp\left(f_{d}\eta +f_{r}\left(V_{E}\right)\eta^{2}\right)=\exp\left\{-j4\pi R\left(\eta ,\zeta_{R}\right)/\lambda \right\}$ is the azimuthal signal without error and $H_{V_{Ee}}(\eta )=\exp(o_{r}\left(V_{Ee}\right)\eta^{2})$ is the error signal caused by the equivalent velocity $V_{Ee}$.

According to Equations (21) and (26), the azimuthal signal with the error can be written as the multiplication between the azimuthal signal without the error and the phase error. Considering the above phase error, the received raw data without the weight $W_{i}\left(\tau_{0},\eta_{n}\right)\cdot T_{r}$ in Equation (2) after range compression and RCMC at a certain range cell is approximately denoted as:

(12) $$s_{error}\left(\tau_{0},\eta \right)=\sum_{i=1}^{M}\sigma_{i}\exp\left\{-j4\pi \frac{R_{i}\left(\eta \right)}{\lambda }\right\}\cdot \exp\left\{j\vartheta \right\}+n\left(\tau_{0},\eta \right)$$

where ϑ is the error phase variation with the azimuthal sampling moment. Similarly to Equation (3), the vector matrix form of the raw signal (27) with an error along the azimuthal sampling moment η is:

(13) $$s_{N\times 1}^{error}=E_{N\times N}D_{N\times M}\sigma_{M\times 1}+n_{N\times 1}$$

where $E_{N\times N}=diag\left(\exp\left(j\vartheta_{1}\right),\exp\left(j\vartheta_{2}\right),\cdots ,\exp\left(j\vartheta_{N}\right)\right)$. A different error has a different expression form of the error matrix $E_{N\times N}$, and the motion error has been not fully formulated yet due to some approximations in the models. Therefore, we assigned an error matrix $E_{N\times N}$ random phase in the following simulation.

### 2.2. Sub-Nyquist SAR Imaging and Error Correction Based on the Pseudo-ℒ_0_-Norm Optimization Algorithm

In this subsection, the CS theorem is first briefly introduced. Then, a pseudo-ℒ_0_-norm regularization model is presented and solved by the Cauchy–Newton method in detail. Finally, sub-Nyquist SAR imaging and error correction based on pseudo-random space-time modulation are proposed based on our proposed pseudo-ℒ_0_-norm optimization algorithm.

#### 2.2.1. CS Theorem

The key to the CS theorem is the effective recovered algorithm. While RIP is satisfied, there are three recovered algorithms, i.e., greedy algorithm, ℒ_1_-norm optimization algorithm, and sparse Bayesian learning method. The ℒ_1_-norm optimization algorithm is robust in the sense that it can effectively recover nearly sparse signals with/without measurement noise from remarkably few measurements, and its application is so wide that it broadly could be considered the modern least squares [12,13]. The ℒ_1_-norm optimization algorithm has a better-recovered performance in terms of the recovered error evaluated by *MSE* [17]. Its optimization equation is to solve the underdetermined problem (3) without a subscript:

(14) $$\hat{\sigma }=\underset{\sigma }{\arg\min}\left\{{\left\|s-D\sigma \right\|}_{2}^{2}+\alpha {\left\|\sigma \right\|}_{1}\right\}$$

where α is the regularization parameter. The first item ${\left\|s-D\sigma \right\|}_{2}^{2}$ ensures the recovery error, and the second item ${\left\|\sigma \right\|}_{1}$ guarantees the sparsity of the recovered scene. The parameter α balances between the recovered error and the sparsity, and is empirically chosen by minimizing the recovered error of the whole scene.

Even though the ℒ_1_-norm optimization algorithm achieved a good recovered performance, some low SNR targets cannot be accurately recovered, and it often yields false targets for an unfair penalization rule. The further optimization of the regularization model contributes to the recovered scene so that false targets can be removed, and then we carried out research about the optimization model. Is there an alternative to the ℒ_1_-norm regularization model to achieve a better-recovered performance? The problem of further improving the optimization algorithm is thus brought up. To solve this problem, a reweighted ℒ_1_-based algorithm has been already proposed to improve the recovered performance [20,21]. However, how to select an approximately fair regularization rule still does not have a theoretical analysis.

#### 2.2.2. Sub-Nyquist SAR Imaging Based on the Pseudo-*L*_0_-Norm Optimization Algorithm

For a sparse undetermined equation, the ℒ_0_-norm optimization algorithm should have the best recovered performance, but this optimization equation is a non-polynomial hard (NP-hard) problem [12]. Based on the idea of adaptive least absolute shrinkage and selection operator (Lasso) technique that is a popular technique for simultaneous estimation and variable selection [21], this paper penalized the regularization item to be close to the ℒ_0_-norm to achieve a better performance of the ℒ_0_-based algorithm, and the regularization model can be solved by the Cauchy–Newton method. Since it is closer to the ℒ_0_-based method in some sense, we named it a pseudo-ℒ_0_-norm optimization algorithm. In the following, we adopted Bayesian estimation to analyze and deduct a pseudo-ℒ_0_-norm regularization model by making full use of the scene prior information. The rule was deducted as follows: Usually, the noise $n_{N\times 1}$ is assumed to be a Gaussian distribution with zero mean and variance ${\sigma_{n}}^{2}$ [14]. For simplicity, all the following matrixes/vectors omit subscripts.

(15) $$p_{n}\left(n\right)=p_{s/\sigma }\left(s/\sigma \right)={\left(\frac{1}{\sqrt{2\pi }\sigma_{n}}\right)}^{N}\exp\left\{-\frac{{\left\|s-D\sigma \right\|}_{2}^{2}}{2{\sigma_{n}}^{2}}\right\}$$

Laplace distribution forces most coefficients to be small so that it can describe the sparse scene [35]. We assume:

(16) $$p\left(\sigma_{i}\right)=\left(\frac{\xi_{i}}{2}\right)\exp\left\{-\xi_{i}{\left\|\sigma_{i}\right\|}_{1}\right\}$$

where $\xi_{i}$ is the scale parameter of Laplace distribution and $\xi_{i}>0$. Then, the probability distribution of the vector $\sigma_{M\times 1}$ is:

(17) $$p_{\sigma }\left(\sigma \right)=\prod_{i=1}^{M}\left\{\left(\frac{\xi_{i}}{2}\right)\exp\left\{-\xi_{i}{\left\|\sigma_{i}\right\|}_{1}\right\}\right\}=\prod_{i=1}^{M}\left\{\left(\frac{\xi_{i}}{2}\right)\right\}\exp\left\{-\sum_{i=1}^{M}\xi_{i}{\left\|\sigma_{i}\right\|}_{1}\right\}$$

Based on the Bayesian rule in the information theory, the maximum posterior (MAP) probability of the vector $\sigma_{M\times 1}$ is:

(18) $$\hat{\sigma }=\arg\max\left[p_{\sigma /s}\left(\sigma /s\right)\right]=\arg\max\left[p_{s/\sigma }\left(s/\sigma \right)\cdot p_{\sigma }\left(\sigma \right)\right]$$

We took the logarithm of the above Formula (18):

(19) $$\begin{array}{ll} \hat{\sigma } & =\underset{\sigma }{\arg\max}\left[\log\left[p_{s/\sigma }\left(s/\sigma \right)\right]+\log\left[p_{\sigma }\left(\sigma \right)\right]\right]\\ & =\arg\max\left[-\frac{1}{2{\sigma_{n}}^{2}}{\left\|s-D\sigma \right\|}_{2}^{2}-{\left\|\xi \cdot \sigma \right\|}_{1}\right]\\ & =\arg\min\left[{\left\|s-D\sigma \right\|}_{2}^{2}+\beta {\left\|\xi \cdot \sigma \right\|}_{1}\right] \end{array}$$

where the reweighting matrix ξ is the diagonal matrix with $\left\{\xi_{1},\xi_{2},\cdots ,\xi_{M}\right\}$ on the diagonal and zeros elsewhere. Similarly to α in Equation (4), β is the regularization parameter. After the above deduction, the regularization model (19) is the modification to the ℒ_1_-norm penalization rule.

Additionally, the matrix ξ is first calculated to solve Equation (19). The logarithm function of the vector $\sigma_{M\times 1}$ is:

(20) $$\begin{array}{l} \log\left\{p_{\sigma }\left(\sigma \right)\right\}=\log\left\{\prod_{i=1}^{M}\left\{\left(\frac{\xi_{i}}{2}\right)\exp\left\{-\xi_{i}{\left\|\sigma_{i}\right\|}_{1}\right\}\right\}\right\}\\ =\log\left\{\prod_{i=1}^{M}\left(\frac{\xi_{i}}{2}\right)\right\}+\log\left\{\exp\left\{-\sum_{i=1}^{M}\xi_{i}{\left\|\sigma_{i}\right\|}_{1}\right\}\right\}=\sum_{i=1}^{M}\log\left(\frac{\xi_{i}}{2}\right)-\sum_{i=1}^{M}\xi_{i}{\left|\sigma_{i}\right|}_{1} \end{array}$$

Let the partial derivative function of Formula (10) with respect to $\xi_{i}$ be equal to zero, and the estimation of the scale parameter $\xi_{i}$ is:

(21) $$\frac{\partial \log\left[p_{\sigma }\left(\sigma \right)\right]}{\partial \xi_{i}}=\frac{1}{\xi_{i}}-{\left\|\sigma_{i}\right\|}_{1}=0$$

(22) $$\xi_{i}=\frac{1}{\left|\sigma_{i}\right|}$$

In the case that $\sigma_{i}=0$, Formula (12) makes no sense. Formula (12) is modified as:

(23) $$\xi_{i}=\frac{1}{\left|\sigma_{i}\right|+\iota }$$

where ι>0 is a very small positive constant.

The above deduction explains the prior distribution, e.g., Laplace distribution in the sub-Nyquist SAR confirms the penalization rule and this method makes full use of the data prior information to achieve a good performance. Certainly, different probability distributions confirm different penalization rules. In the scenario of not knowing the variables themselves, using the iteratively updated method to establish an approximately fair penalization rule allows for the successively better estimation of nonzero variables. According to the optimization Equations (19) and (23), we know that the regularized item is iteratively penalized by itself to nearly acquire the number of nonzero values, and the large coefficients are more heavily penalized to discourage their effects compared to that with a small coefficient and is more likely to be identified as nonzero. Once the nonzero locations are identified, their influence attenuates to allow more sensitivity to identify the remaining small but nonzero elements. It means this algorithm can more accurately recover low SNR targets.

To solve the pseudo-ℒ_0_-norm regularization equation, the algorithm in Equation (19) is essentially a ℒ_1_ penalization method so that it is convex and can be solved by the Cauchy–Newton method [36,37]. The solving method is as follows: ℒ_1_ norm is not differentiable, while $\sigma_{i}=0$. Firstly, the smoothing approximation is introduced:

(24) $${\left\|\sigma \right\|}_{1}=\sum_{i=1}^{N}\sqrt{\left({\left|\sigma_{i}\right|}^{2}+\varsigma \right)}$$

where ς>0 is a very small positive constant. Equation (19) can be expressed as:

(25) $$\hat{\sigma }=\underset{\bar{\sigma }}{\arg\min}\left\{{\left\|s-D\sigma \right\|}_{2}^{2}+\beta \sum_{i=1}^{M}\xi_{i}\sqrt{\left({\left|\sigma_{i}\right|}^{2}+\varsigma \right)}\right\}$$

where $f\left(\hat{\sigma }\right)={\left\|s-D\sigma \right\|}_{2}^{2}+\beta \sum_{i=1}^{M}\xi_{i}\sqrt{\left({\left|\sigma_{i}\right|}^{2}+\varsigma \right)}$. The conjugate gradient function is written as:

(26) $$\nabla_{\hat{\sigma }}f\left(\hat{\sigma }\right)=2D^{H}D\sigma +\beta U\left(\hat{\sigma }\right)\cdot \xi \sigma -2D^{H}s=H\left(\hat{\sigma }\right)\sigma -2D^{H}s$$

where $H\left(\hat{\sigma }\right)=2D^{H}D+\beta U\left(\hat{\sigma }\right)\xi$, $U\left(\hat{\sigma }\right)=diag\left\{1/\left(\sqrt{{\left|\sigma_{i}\right|}^{2}+\varsigma }\right)\right\},i=1,2,\cdots ,M$. $D^{H}$ is the conjugate transpose of the observed matrix D.

According to the Newton method [36,37], the iterative equation of the reconstructed result σ is:

(27) $$H\left({\hat{\sigma }}^{\left(g\right)}\right){\hat{\sigma }}^{\left(g+1\right)}=\left(1-\gamma \right)H\left({\hat{\sigma }}^{\left(g\right)}\right){\hat{\sigma }}^{\left(g\right)}+2\gamma D^{H}s$$

where γ is the step size of the iteration. When γ=1, Equation (27) is simplified as:

(28) $${\hat{\sigma }}^{\left(g+1\right)}=2H{\left({\hat{\sigma }}^{g}\right)}^{-1}D^{H}s$$

Until the iteration terminates, $\hat{\sigma }$ is the recovered result. Normally, the *MSE*, the iterative number, etc., can be taken as the iterative criterion [23]. After the above deduction, the flow chart is concluded as follows:

(1) Initialization: the iterative step g=1, ${\hat{\sigma }}^{1}=D^{H}s$;
(2) Updating of the weighting matrix ξ and the matrix $H\left(\hat{\sigma }\right)$: $\xi^{\left(g\right)}=diag\left(\frac{1}{\left|\sigma_{1}\right|+\iota },\frac{1}{\left|\sigma_{2}\right|+\iota },\cdots ,\frac{1}{\left|\sigma_{M}\right|+\iota }\right)$, $H{\left(\hat{\sigma }\right)}^{\left(g\right)}=2D^{H}D+\beta U\left(\hat{\sigma }\right)\xi^{\left(g\right)}$;
(3) Calculation: ${\hat{\sigma }}^{\left(g+1\right)}=2{\left(H{\left(\hat{\sigma }\right)}^{\left(g\right)}\right)}^{-1}D^{H}s$;
(4) g=g+1;
(5) Loop;
(6) Stopping iteration according to the iterative criterion.

During the deduction of the pseudo-ℒ_0_-norm optimization algorithm, there are four undetermined parameters, i.e., the regularization factor β, the parameter ι, the parameter ς, and the iterative criterion. Similarly to the regularization α, β is also an empirical value and is chosen by minimizing the recovered error [12]. In the simulation section, the iterative criterion selects the preset iterative number and this number is chosen empirically. Although the reference [23] demonstrates how to choose the parameter ι and ς, the pseudo-ℒ_0_-norm optimization algorithm has a good robustness and the reconstructed scene does have not the strictly sparse property; so, the proposed algorithm sets the parameters ι and ς as $10^{-3}$ and $10^{-6}$, respectively.

#### 2.2.3. Error Correction Based on the Pseudo-ℒ_0_-Norm Optimization Algorithm

Based on the observation model of Equation (13), we propose an error correction method integrated with the pseudo-ℒ_0_-norm optimization algorithm. This method considers the phase error as the model error and removes it during the scene reconstruction, and it enables our method to correct more artifacts due to the robustness of the pseudo-ℒ_0_-norm optimization algorithm. This error correction method is an update and iteration algorithm, and it includes the scene reconstruction and the error estimation during each iteration. In the first step of every iteration, the cost function is minimized with the scene based on the reweighted ℒ_1_-norm optimization algorithm, and in the second step, the phase error is estimated given the scene estimate. According to the estimated error, the error matrix $E_{N\times N}$ in (13) is updated and delivered to the next iteration. The procedure is as follows:

(1) The scene reconstruction

Based on the pseudo-ℒ_0_-norm optimization algorithm, the optimization equation of (13) without subscripts is:

(29) $$\hat{\sigma }=\underset{\sigma }{\arg\min}\left[{\left\|s^{error}-ED\sigma \right\|}_{2}^{2}+\beta {\left\|\xi \sigma \right\|}_{1}\right]$$

The flow chart in Section 2.2.2 can resolve this optimization equation.

(2) The error estimation

To estimate the phase error, the cost function should minimize the *MSE* of the reconstructed result since the error exists at each sampling moment, and each element can be handled separately. The cost function at each sampling moment is:

(30) $${\hat{E}}_{n}\left(g+1\right)=\underset{E_{n}}{\arg\min}{{\left\|s_{n}^{error}-E_{n}{\left(D^{\left(g\right)}\sigma^{\left(g+1\right)}\right)}_{n}\right\|}_{2}}^{2},n\in \left\{1,2,\cdots ,N\right\}$$

where ${\hat{E}}_{n}\left(g+1\right)$ is the *n*-th diagonal element of the error matrix ${\hat{E}}^{\left(g+1\right)}$ at the (*g* + 1)-th iteration. In the following, the cost function is unfolded for analysis:

(31) snerror−EnDgσg+1n22=snerror−EnDgσg+1nH⋅snerror−EnDgσg+1n=snerrorH⋅snerror+Dgσg+1nH⋅Dgσg+1n−2cosϑn⋅ResnerrorH⋅Dgσg+1n+2sinϑn⋅ImsnerrorH⋅Dgσg+1n

where Re· and Im· are the real part and the imaginary part of a complex signal, respectively. Assuming that ρ=ResnerrorH⋅Dgσg+1n and υ=ImsnerrorH⋅Dgσg+1n, the above equation can be written as:

(32) $${{\left\|s_{n}^{error}-E_{n}{\left(D^{\left(g\right)}\sigma^{\left(g+1\right)}\right)}_{n}\right\|}_{2}}^{2}={\left(s_{n}^{error}\right)}^{H}\cdot s_{n}^{error}+{{\left(D^{\left(g\right)}\sigma^{\left(g+1\right)}\right)}_{n}}^{H}\cdot {\left(D^{\left(g\right)}\sigma^{\left(g+1\right)}\right)}_{n}-2\sqrt{\rho^{2}+\upsilon^{2}}\cdot \cos\left(\vartheta_{n}-\arctan\left(\frac{\upsilon }{\rho }\right)\right)$$

Estimating $\vartheta_{n}$ is equal to minimizing the above cost function (32); so, ${\hat{\vartheta }}_{n}$ satisfies:

(33) $${\hat{\vartheta }}_{n}=∠{\left(s_{n}^{error}\right)}^{H}\cdot {\left(D^{\left(g\right)}\sigma^{\left(g+1\right)}\right)}_{n}$$

After the above deduction, the detailed flow chart is as follows:

(1) Initialization: g=0, E=I;
(2) Recovering the scene: ${\hat{\sigma }}^{\left(g\right)}=\underset{\sigma }{\arg\min}{\left\|s^{error}-E^{\left(g\right)}D\sigma^{\left(g\right)}\right\|}_{2}+\beta {\left\|\xi^{\left(g\right)}\sigma^{\left(g\right)}\right\|}_{1}$;
(3) Estimating the error matrix: ${\hat{E}}^{\left(g\right)}=\underset{E}{\arg\min}{\left\|s^{error}-E^{\left(g\right)}D\sigma^{\left(g\right)}\right\|}_{2}$;
(4) Updating $E^{\left(g\right)}$;
(5) g=g+1;
(6) Loop;
(7) Stopping iteration according to the iterative criterion.

where ${\hat{\sigma }}^{\left(g\right)}$ and ${\hat{E}}^{\left(g\right)}$ denote the recovered result and the estimated error matrix in the *g*-th iteration, respectively. During each iteration, the cost function focuses part of the defocused scene to generate a relatively accurate error matrix so that a relatively accurate model can lead to a better reconstruction result. The iterative criterion was mentioned in Section 2.2.2 and is not repeated in this section.

#### 2.2.4. Analysis of the Computational Complexity

In order to obtain the total computational complexity of the proposed method, we first analyzed the time complexity per iteration quantitatively. As the notations used above, the total computational cost of the proposed method is in the order of $ο\left(G\cdot NM\log\left(NM\right)\right)$, where G is the required number of iterations to recover the result in one range cell. The value of G is difficult to confirm accurately through theoretical analysis, but in practice, the running time of the proposed method is affordable. As for the memory cost of the proposed method, we only need to store the input, the output, and the parameter matrices. In summary, the memory cost is in the order of $ο\left(NM\right)$.

## 3. Experiment

In this section, simulation experiments and data experiments verify the effectiveness and superiority of sub-Nyquist SAR imaging and error correction integrated with a pseudo-ℒ_0_-norm optimization algorithm.

### 3.1. Data Description

To verify the validity and effectiveness of sub-Nyquist SAR imaging and error correction based on the pseudo-ℒ_0_-norm optimization algorithm, we selected real strip-map TerraSAR-X images for the experiments. The reflectivity functions of images were used to simulate raw data according to the simulated parameters, and then the raw data were uniformly received, as shown in Figure 1. During the error correction, the error is added at each sampling moment and the error adopts the random distribution.

**Figure 3:**
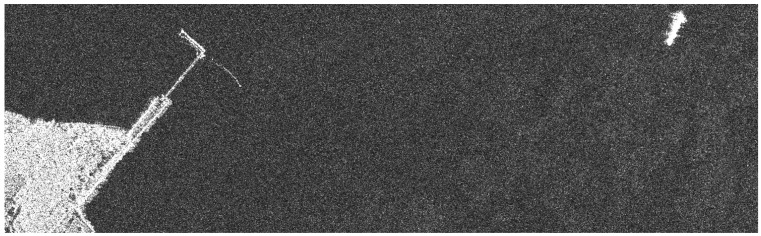
Sea–land interface scene in the SAR image.

**Figure 4:**
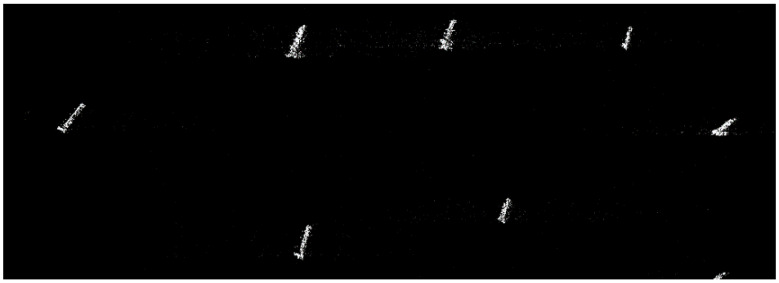
Sea containing several boats in the SAR image.

Figure 3 features a sea–land interface scene. This scene is more complex and used to verify the performance of our proposed algorithm in the sub-Nyquist SAR imaging compared with the ℒ_1_-norm optimization algorithm. Figure 4 is a sea containing several boats to verify the availability of error corrections based on the pseudo-ℒ_0_-norm optimization algorithm.

### 3.2. The Simulation of the Pseudo-ℒ_0_-Norm Optimization Algorithm

We used a one-dimensional simulation of point targets to verify the superiority of our proposed algorithm compared with the ℒ_1_-norm optimization algorithm. For a given under-recovered signal, σ, the recovered performance can be evaluated by the normalized *MSE (NMSE)*:

(34) $$NMSE=\frac{{\left\|\hat{\sigma }-\sigma \right\|}_{2}^{2}}{{\left\|\sigma \right\|}_{2}^{2}}$$

where $\hat{\sigma }$ is the recovered result.

**Figure 5:**
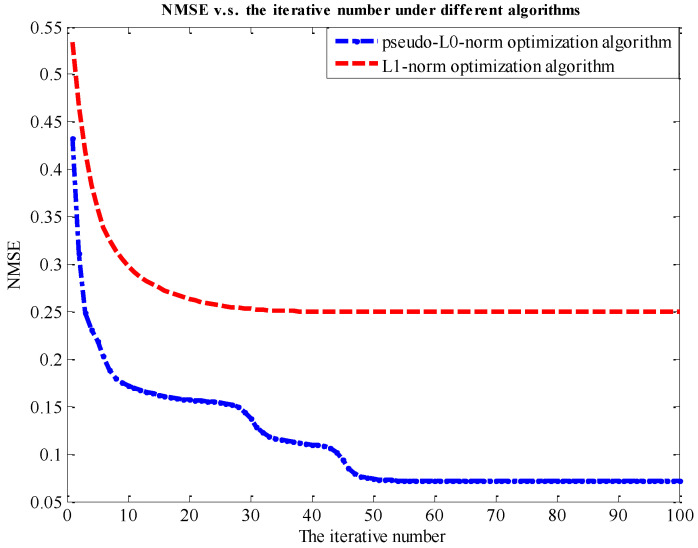
*NMSE* vs. the iterative number under different algorithms.

**Figure 6:**
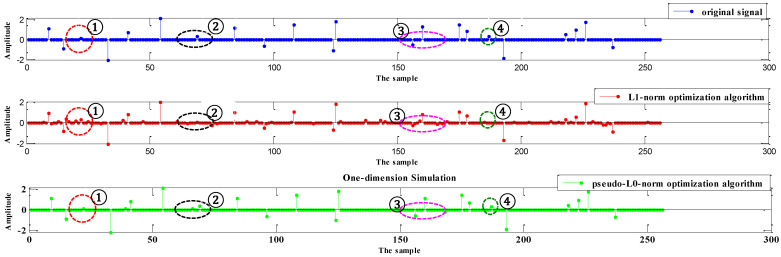
The recovered result under different algorithms.

The following one-dimensional simulation illustrates that the pseudo-ℒ_0_-norm algorithm has a better performance compared with the ℒ_1_-norm optimization algorithm. The simulated parameters are as follows. It selects a sparse signal, σ, of length M=256, with ${\left\|\sigma \right\|}_{0}=S$. The S nonzero spike positions are selected randomly, and the amplitude of nonzero elements obeys a zero-mean unit-variance Gaussian distribution. This simulation selects the measured number and a N×M random matrix, D, with independent identically distributed (i.i.d.) Gaussian elements. The noise vector, n, is drawn from the i.i.d. zero-mean Gaussian function with ${\left\|n\right\|}_{2}=0.5$, so that $SNR=10log10({\left\|\sigma \right\|}_{2}^{2}/{\left\|n\right\|}_{2}^{2})=21dB$. To recover the signal, σ, it adopts two algorithms, the ℒ_1_-norm optimization algorithm and pseudo-ℒ_0_-norm algorithm, to compare the recovered performance. The *NMSE* of the two algorithms and the recovered results are compared in Figure 5 and Figure 6.

Figure 5 demonstrates that the pseudo-ℒ_0_-norm optimization algorithm can achieve the smallest recovered error, although this proposed algorithm has a slower rate of convergence. Our proposed method is sensitive to low SNR targets that are not easy to identify, so the convergence rate is slow. Figure 6 shows the recovered results under different algorithms. In the red circle, ①, the results for the ℒ_1_-norm optimization algorithm have weak false targets, but two other algorithms do not. In circles ② and ④, the weak target can be recovered under the optimization algorithm, while the ℒ_1_-norm optimization algorithm cannot recover it. In circle ③, the amplitude of targets can be exactly recovered by the proposed algorithm. It demonstrates that our proposed algorithm is more friendly to the low SNR target and removes false targets to achieve a good recovered performance.

### 3.3. The Simulation of Sub-Nyquist SAR Imaging

**Figure 7:**
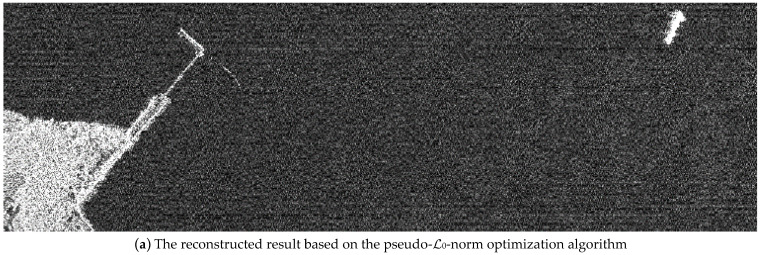

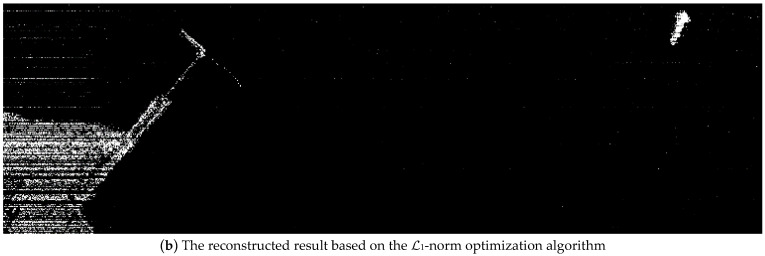
The recovered result based on the pseudo-ℒ_0_-norm optimization algorithm and ℒ_1_-norm optimization algorithm.

We used a simulation based on the real strip-map TerraSAR-X image in Figure 3 to verify the validity of our proposed algorithm for sub-Nyquist SAR imaging. The SAR image is over the sea–land interface scene. The simulated parameters are shown in Table 1, and the raw data were randomly received, as shown in Figure 1. In the simulation, the interfaced land is recovered without the loss of details under the proposed algorithm. According to the simulated result in Figure 7 and *MSE* in Table 2, this simulation also demonstrates that the pseudo-ℒ_0_-norm optimization algorithm can achieve a better recovered performance compared with the ℒ_1_-norm optimization algorithm.

### 3.4. Simulation of the Error Correction

The error correction mainly solves image defocusing for the phase error. As we all know, the better the focusing performance is, the smaller the image entropy is. We adopted image entropy $H\left(\sigma \right)$ to evaluate the reconstruction result.

(35) $$H\left(\sigma \right)=-\sum_{i}\frac{{\left|\sigma_{i}\right|}^{2}}{\sum_{i}{\left|\sigma_{i}\right|}^{2}}\log_{2}\frac{{\left|\sigma_{i}\right|}^{2}}{\sum_{i}{\left|\sigma_{i}\right|}^{2}}$$

**Figure 8:**
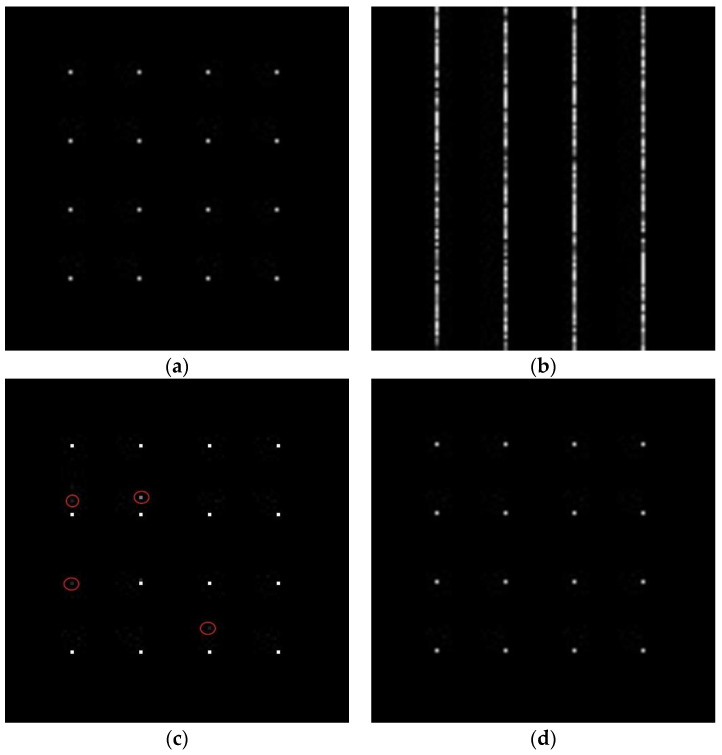
The reconstructed results. (**a**) The original image; (**b**) the reconstructed scene without error correction; (**c**) the reconstructed scene with error correction based on the ℒ_1_-norm optimization algorithm; and (**d**) the reconstructed scene with error correction based on the pseudo-ℒ_0_-norm optimization algorithm.

Firstly, we simulated point targets to illustrate the effectiveness of our method. The simulated parameters are shown in Table 3. When the elements of the error matrix are uniformly distributed $\left[0,17/18\pi \right]$, the simulated results are presented in Figure 8. Figure 8a is the original image, (b) is the reconstructed scene without an error correction in the traditional SAR, (c) is the reconstructed scene with an error correction in the sub-Nyquist SAR based on the ℒ_1_–norm optimization algorithm, and (d) is the reconstructed scene with the error correction in the sub-Nyquist SAR based on the pseudo-ℒ_0_-norm optimization algorithm. When the phase error exists, the reconstructed scene without an error correction is defocused (Figure 8b). Although it can also remove errors and focus the scene based on the ℒ_1_-norm optimization algorithm, there are false targets and lost targets in the red circle (Figure 8c). Our method almost removes errors and recovers the scene based on the pseudo-ℒ_0_-norm optimization algorithm (Figure 8d). From the quantitative analysis, Table 4 illustrates that the image entropy based on our method achieves the nearly same image entropy as the original image.

**Figure 9:**
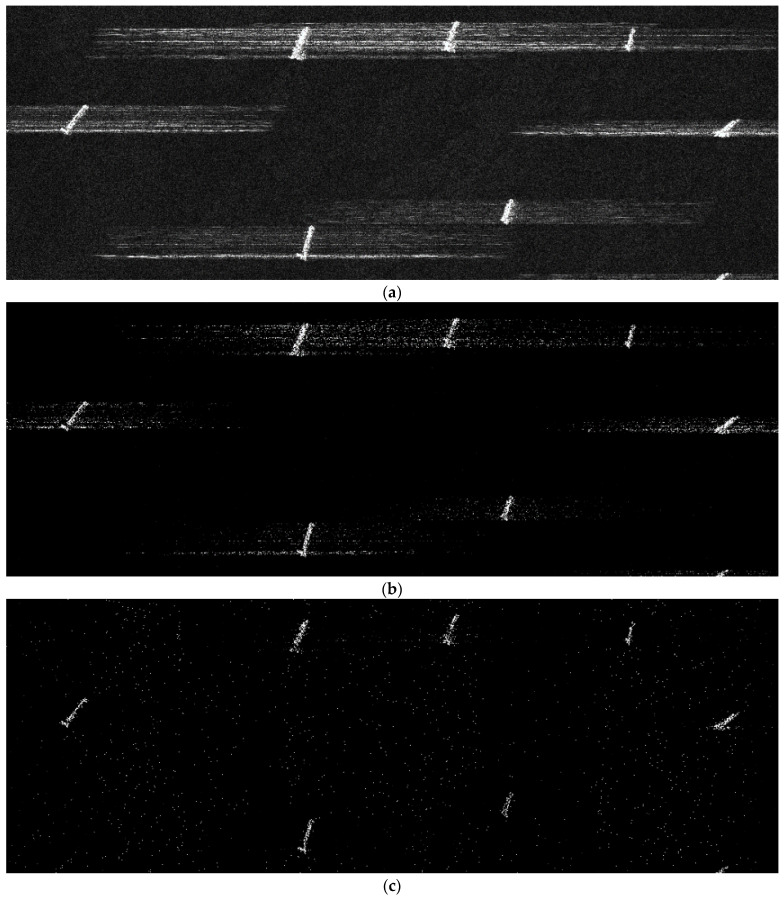
The reconstructed result. (**a**) The scene reconstruction without error correction; (**b**) the scene reconstruction with error correction based on the ℒ_1_-norm optimization algorithm; and (**c**) the scene reconstruction with error correction based on the pseudo-ℒ_0_-norm optimization algorithm.

The simulation in Figure 9 is based on a strip-map TerraSAR-X image, i.e., sea containing several boats in Figure 4. The reflectivity function of the image was used to simulate raw data, and the simulated parameters are shown in Table 3. The elements of the error matrix are uniformly distributed, $\left[0,\pi /2\right]$. Figure 9a is the recovered result in the traditional SAR, but is defocused for the phase error. The error is partly removed from the Sub-Nyquist SAR with an error correction method based on the ℒ_1_-norm optimization algorithm in Figure 9b. Based on the pseudo-ℒ_0_-norm optimization algorithm, it almost removes the error and focuses the scene in Figure 9c. The image entropy in Table 5 illustrates that the image recovered by our method can achieve a similar performance to the original image.

## 4. Discussion

HRWS SAR imaging has always been the goal of spaceborne SAR systems in remote sensing applications [2]. Since a high resolution and wide swath are inherently conflicting requirements, the HRWS SAR system creates new difficulties and challenges. These requirements are simultaneously satisfied by advanced observing modes, e.g., azimuthal multi-channel SAR [3] and MIMO SAR [4], and these observing modes both have the characteristics of large amounts of data and long antenna. As the CS theorem develops, a novel imaging mode named sub-Nyquist SAR based on the pseudo-random space–time modulation has been proposed without large data and long antenna, and sub-Nyquist SAR imaging based on the CS algorithms can recover the scene [8].

Although the ℒ_1_-norm optimization algorithm has achieved a quite good performance, some low SNR targets are not accurately recovered and it yields false targets. To further improve the recovered performance of sub-Nyquist SAR, we present a pseudo-ℒ_0_-norm optimization algorithm based on the Bayesian estimation. This algorithm penalizes the regularization item with the reciprocal of its previous solution to acquire nonzero values so that the rule is fair and makes full use of the data prior information. This proposed algorithm essentially adopts the idea of the adaptive Lasso technique [20,21], and the ℒ_1_-norm optimization algorithm is the Lasso variable selection method [13]. The Lasso variable selection is consistent with satisfying a necessary condition; sometimes Lasso is not consistent and does not have predictive properties. Adaptive Lasso is the modified version of the Lasso system with important differences. The modification is data-dependent and reasonably selected so that adaptive Lasso has predictive properties [20,21]. Our proposed algorithm is better in terms of consistency. For the recovered error, we deducted the expression of *MSE* in the following way. Assume that $F_{N\times S}$ is the submatrix constructed by taking S columns from the recovered matrix, $D_{N\times M}$, which are specified by the index vector, Λ, and each element in Λ satisfies the following:

(36) $$\sigma_{\Lambda_{l}}\neq 0,\left(l=1,2,\cdots ,S\right)$$

The estimated covariance matrix for the nonzero components, $\sigma_{l}$, is

(37) $$C={\left({F_{N\times S}}^{H}F_{N\times S}+\gamma_{2}\Sigma \left(\sigma_{\Lambda_{l}}\right)\right)}^{-1}{F_{N\times S}}^{H}F_{N\times S}{\left({F_{N\times S}}^{H}F_{N\times S}+\gamma_{2}\Sigma \left(\sigma_{\Lambda_{l}}\right)\right)}^{-1}$$

where $\Sigma \left(\sigma_{\Lambda_{l}}\right)=diag\left(\xi_{\Lambda_{1}}/\sigma_{\Lambda_{1}},\xi_{\Lambda_{2}}/\sigma_{\Lambda_{2}},\cdots ,\xi_{\Lambda_{S}}/\sigma_{\Lambda_{S}}\right)$ is a diagonal matrix. The estimated error is the trace of the covariance matrix, C:

(38) $$\begin{array}{l} {\left\|{\hat{\sigma }}_{\Lambda }-\sigma_{\Lambda }\right\|}_{2}^{2}={\sigma_{n}}^{2}trace\left(C\right)\\ ={\sigma_{n}}^{2}trace\left({\left({F_{N\times S}}^{H}F_{N\times S}+\gamma_{2}\Sigma \left(\sigma_{\Lambda_{l}}\right)\right)}^{-1}{F_{N\times S}}^{H}F_{N\times S}{\left({F_{N\times S}}^{H}F_{N\times S}+\gamma_{2}\Sigma \left(\sigma_{\Lambda_{l}}\right)\right)}^{-1}\right)\\ ={\sigma_{n}}^{2}trace\left({\left({\left({F_{N\times S}}^{H}F_{N\times S}+\gamma_{2}\Sigma \left(\sigma_{\Lambda_{l}}\right)\right)}^{-1}\right)}^{2}{F_{N\times S}}^{H}F_{N\times S}\right)\\ \geq {\sigma_{n}}^{2}\left[2trace\left({\left({F_{N\times S}}^{H}F_{N\times S}+\gamma_{2}\Sigma \left(\sigma_{\Lambda_{l}}\right)\right)}^{-1}\right)-trace\left({\left({F_{N\times S}}^{H}F_{N\times S}\right)}^{-1}\right)\right] \end{array}$$

Assuming that $A={F_{N\times S}}^{H}F_{N\times S}$, $B={F_{N\times S}}^{H}F_{N\times S}+\gamma_{2}\Sigma \left(\sigma_{\Lambda_{l}}\right)$, and their eigenvalues are $\lambda_{1},\lambda_{2},\cdots ,\lambda_{S}$ and $\alpha_{1},\alpha_{2},\cdots ,\alpha_{S}$, respectively. Based on the structure form of the recovered matrix, $D_{N\times M}$, the ℒ_2_-norm of each column is nearly equal and assumes ${\left\|D_{l}\right\|}_{2}^{2}\approx L$, where L is the number of samples in one aperture time [8]. Formula (38) is simplified as:

(39) $${\left\|{\hat{\sigma }}_{\Lambda }-\sigma_{\Lambda }\right\|}_{2}^{2}\geq {\sigma_{n}}^{2}\left[2trace\left(B^{-1}\right)-trace\left(A^{-1}\right)\right]$$

Gail’s circle theorem [38] indicates that

(40) $$\begin{array}{c} \left|\lambda_{l}-L\right|\leq \left(S-1\right)\cdot u\cdot L\\ \left(1-\left(S-1\right)\cdot u\right)\cdot L\leq \lambda_{l}\leq \left(1+\left(S-1\right)\cdot u\right)\cdot L\\ \frac{1}{\left(1+\left(S-1\right)\cdot u\right)\cdot L}\leq {\lambda_{l}}^{-1}\leq \frac{1}{\left(1-\left(S-1\right)\cdot u\right)\cdot L}\\ \frac{S}{\left(1+\left(S-1\right)\cdot u\right)\cdot L}\leq \sum_{l=1}^{S}{\lambda_{l}}^{-1}\leq \frac{S}{\left(1-\left(S-1\right)\cdot u\right)\cdot L}\\ \frac{S}{\left(1+\left(S-1\right)\cdot u\right)\cdot L}\leq trace\left(A^{-1}\right)\leq \frac{S}{\left(1-\left(S-1\right)\cdot u\right)\cdot L} \end{array}$$

(41) αl−L+γ2⋅ξΛlσΛl≤S−1⋅u⋅LσΛl⋅1−S−1⋅u⋅L+γ2⋅ξΛlσΛl≤αl≤σΛl⋅1+S−1⋅u⋅L+γ2⋅ξΛlσΛlσΛlσΛl⋅1+S−1⋅u⋅L+γ2⋅ξΛl≤αl−1≤σΛlσΛl⋅1−S−1⋅u⋅L+γ2⋅ξΛlS1+S−1⋅u⋅L+γ2⋅maxξΛlσΛl≤∑l=1Sαl−1≤S1−S−1⋅u⋅L+γ2⋅minξΛlσΛlS1+S−1⋅u⋅L+γ2⋅maxξΛlσΛl≤traceB−1≤S1−S−1⋅u⋅L+γ2⋅minξΛlσΛl

where $u=\underset{1\leq m_{1}\neq m_{2}\leq M}{\max}\left|\langle D_{m_{1}},D_{m_{2}}\rangle \right|/\left({\left\|D_{m_{1}}\right\|}_{2}\cdot {\left\|D_{m_{2}}\right\|}_{2}\right)$ is the mutual coherence coefficient that reflects the maximum similarity between any two different columns, $m_{1}$, $m_{2}$, in the recovered matrix, $D_{N\times M}$. Combine Formulas (40) and (41):

(42) 2S1+S−1⋅u⋅L+γ2⋅maxξΛlσΛl−S1−S−1⋅u⋅L≤2traceB−1−traceA−1≤2S1−S−1⋅u⋅L+γ2⋅minξΛlσΛl−S1+S−1⋅u⋅L

So, we have the *MSE* of the proposed algorithm:

(43) σ^Λ−σΛ22≥σn22traceB−1−traceA−1≥σn22S1+S−1⋅u⋅L+γ2⋅maxξΛlσΛl−S1−S−1⋅u⋅L

Similarly, the *MSE* of the ℒ_1_-norm optimization algorithm is:

(44) σ^l1−σΛ22≥σn22traceB−1−traceA−1≥σn22S1+S−1⋅u⋅L+γ1⋅max1σΛl−S1−S−1⋅u⋅L

From the above deduction, we can see the optimal performance is achieved by the two above algorithms. By comparing (43) and (44), it can be observed that the difference is in the dashed block. The pseudo-ℒ_0_-norm optimization algorithm is more sensitive to small coefficients. For the low SNR targets, i.e., $\xi_{\Lambda_{l}}=1/\left(\sigma_{\Lambda_{l}}+\iota \right)≻1$, our proposed algorithm performs better and it can more accurately recover low SNR targets. For example, in the one-dimensional simulation, our algorithm recovers the weak target, and the amplitude of the target is more accurately recovered in Figure 6.

Sub-Nyquist SAR imaging includes three steps: range compression, range cell migration correction (RCMC), and azimuth compression. After range compression and RCMC, a pseudo-ℒ_0_-norm optimization algorithm is employed to achieve the azimuth compression. Simulation experiments in Figure 5 and Figure 6, and data experiments based on a real TerraSAR-X image in Figure 7, demonstrate that the algorithm can achieve smaller recovered errors and remove false targets compared to that of the ℒ_1_-norm optimization algorithm. Additionally, considering that CS algorithms themselves have a certain capacity to remove phase errors, an error correction method integrated with a pseudo-ℒ_0_-norm optimization algorithm eliminates the influence of phase errors and removes defocusing. Different from autofocusing technology as a post-processing method in a traditional SAR system, this method includes two steps: scene reconstruction based on a pseudo-ℒ_0_-norm optimization algorithm and error estimation by minimizing the least-square target function. These two steps are successively iterative and the recovered matrix is updated to the estimated error. Since the exact expression of the error is not fully formulated, we propose the error matrix with a random phase, whatever the expression for the error. The simulations in the Figure 8 and Figure 9 explain the advantages of our proposed algorithm.

The potential applications of the pseudo-ℒ_0_-norm optimization algorithm can recover more low SNR targets, e.g., the sea–land interface in the above experiment, so that it is not limited to high SNR targets, e.g., the boats on the sea. This algorithm can also be applied to the traditional HRWS system, e.g., the azimuthal multi-channel SAR and MIMO SAR, to lower the amount of data to relieve the pressure on data storage.

## 5. Conclusions

In this paper, we propose a signal processing algorithm for an innovative single-channel HRWS system called sub-Nyquist SAR based on a pseudo-random space–time modulation. To further improve sub-Nyquist SAR imaging performance, a pseudo-ℒ_0_-norm optimization algorithm is proposed. Firstly, the Bayesian estimation explains how to take a more democratic approach with data prior information to acquire nonzero variables, so that it can more accurately recover low SNR targets and remove false targets compared with the prevalent ℒ_1_-norm optimization algorithm. Then we present an error correction method integrated with a pseudo-ℒ_0_-norm optimization algorithm to eliminate the effect of phase errors and achieve autofocusing. Finally, the simulated experiments demonstrate the effectiveness of the proposed algorithm.

## Figures and Tables

**Table 1:** Simulated parameters.

Parameter	Data
Average PRF (Hz)	893
Range sampling frequency (MHz)	55
Referred slant range (km)	870
Chirp rate (Hz/s)	10^12^
Doppler bandwidth (Hz)	2438
Wavelength (mm)	5.55
Velocity (m/s)	7513
Height (km)	693
Squint angle (°)	0

**Table 2:** *MSE* under different algorithms.

	pseudo-ℒ_0_-norm optimization algorithm	ℒ_1_-norm optimization algorithm
*MSE*	0.114	0.874

**Table 3:** Simulated parameters.

Parameter	Data
Average PRF (Hz) in the sub-Nyquist SAR	155
PRF (Hz) in the traditional SAR	1907
Range sampling frequency (MHz)	120
Referred slant range (km)	888
Pulse width (us)	50
Doppler bandwidth (Hz)	1401
Wavelength (mm)	5.55
Velocity (m/s)	7513
Height (km)	693
Squint angle (°)	0

**Table 4:** Image entropy under different algorithms.

	The original image	The reconstructed scene without an error correction	The reconstructed scene with an error correction based on the ℒ_1_-norm optimization algorithm	The reconstructed scene with an error correction based on the pseudo-ℒ_0_-norm optimization algorithm
Image entropy (*bit*)	4.00	5.90	3.08	3.99

**Table 5:** Image entropy under different algorithms.

	The original image	The reconstructed scene without an error correction	The reconstructed scene with an error correction based on the ℒ_1_-norm optimization algorithm	The reconstructed scene with an error correction based on the pseudo-ℒ_0_-norm optimization algorithm
Image entropy (bit)	7.81	12.16	8.35	7.80

## Data Availability

Restrictions apply to the availability of these data. Data were bought from Deutsches Zentrum für Luft- und Raumfahrt (DLR) and are available from the author Wenjiao Chen with the permission of DLR.
